# Mycotoxin Identification and In Silico Toxicity Assessment Prediction in Atlantic Salmon

**DOI:** 10.3390/md18120629

**Published:** 2020-12-10

**Authors:** Josefa Tolosa, Francisco J. Barba, Noelia Pallarés, Emilia Ferrer

**Affiliations:** Laboratory of Food Chemistry and Toxicology, Faculty of Pharmacy, University of Valencia, Avenue Vicent Andrés Estellés s/n, 46100 Burjassot, Spain; Josefa.tolosa@uv.es (J.T.); Noelia.pallares@uv.es (N.P.); emilia.ferrer@uv.es (E.F.)

**Keywords:** liquid chromatography, time of flight mass spectrometry, mycotoxins, Atlantic salmon, in silico prediction

## Abstract

The present study aimed to identify mycotoxins in edible tissues of Atlantic salmon (*Salmo salar*) using liquid chromatography coupled to hybrid quadrupole time-of-flight mass spectrometry (LC-Q-TOF-MS). After using a non-targeted screening approach and a home-made spectral library, 233 mycotoxins were analyzed. Moreover, the occurrence of mycotoxins in fish filets was evaluated, and their potential toxicity was predicted by in silico methods. According to the obtained results, forty mycotoxins were identified in analyzed salmon samples, the predominant mycotoxins being enniatins (also rugulosin and 17 ophiobolins), commonly found in cereals and their by-products. Thus, mycotoxin carry-over can occur from feed to organs and edible tissues of cultivated fish. Moreover, the toxicity of detected mycotoxins was predicted by the in silico webserver ProTox-II, highlighting that special attention must be paid to some less reported mycotoxins due to their toxic predicted properties.

## 1. Introduction

Mycotoxins are natural contaminants commonly found in plant-derived foodstuffs, mainly cereals and their by-products. Since these raw materials are added as ingredients in feed formulation for different animal species, including cultivated fish, the risk of mycotoxin contamination in feed for aquaculture has increased, thus introducing contaminants (i.e., mycotoxins), which were not previously identified in fish tissues [1]. Diverse studies reported mycotoxin contents in a wide range of randomly sampled feedstuffs and raw materials intended for terrestrial animals [2,3,4,5,6,7]. However, studies focused on feedstuffs intended for aquaculture fish are still scarce, although recently, some studies developed feasible analytical approaches for mycotoxin detection in aquafeeds [8,9]. The carry-over of mycotoxins from feed into edible portions of fish indicate that mycotoxins and their metabolites present in raw materials and feed for aquaculture fish can be fixed in edible portions and organs [10,11,12].

In addition, mycotoxins have the ability to enter into the food chain through the intake of animal derived products such as milk, meat and eggs from livestock and poultry fed with contaminated feed. Some studies stated that the exposure risk to humans by consumption of these animal derived products can be considered as negligible due to lower contents reported in most cases [12,13]. However, it should be highlighted that mycotoxins or their metabolites can be considered an additional risk to human health, since they are part of the diet in combination with other chemical contaminants. Moreover, the exposure risk derived from the consumption of these animal by-products also depends on other factors, such as the considered diet, different groups of consumers with different metabolic profiles and their health status.

Mycotoxins have an important impact on aquaculture farming. However, there is a lack of information regarding the consequences for reared fish species, especially compared to that on terrestrial species [14]. Therefore, due to growing expansion of aquaculture feedstuffs, there is a need to control mycotoxin occurrence in fish produced by this production sector, since more data are required to carry out an adequate risk assessment for human consumption.

Atlantic salmon (*Salmo salar*) is among the most important farmed fish in Europe, together with other species such as rainbow trout (*Oncorhynchus mykiss*), sea bass (*Dicentrarchus labrax*) and gilthead sea bream (*Sparus aurata*). These species have been the key to producing an increase in the demand and fish consumption and production, thus converting their capture into their aquaculture farming [15]. The European Commission established a maximum level (ML) for aflatoxin M1 (AFM1) in milk [16]. However, no maximum levels (MLs) have been set for other mycotoxins in animal source foods (ASF), due to the scarce information on their occurrence in these foodstuffs [17,18]. Nevertheless, MLs have been set for AFB1 in feed and raw materials, while for deoxynivalenol (DON), zearalenone (ZEA), ochratoxin A (OTA), fumonisins (FB1 and FB2), HT-2 and T-2 toxins, maximum thresholds have been recommended in European legislation. However, according to Bernhoft et al. (2018) [11], the maximum recommended level of DON is inappropriate as in their study, the calculated Non-Observed Adverse Effect Level (NOAEL) was lower than the guidance value.

Within this context, multi-mycotoxin methods have been developed in order to investigate the mycotoxin levels in feedstuffs and thus to assess the carry-over from feed to edible tissues [7,19]. These methods employing mass spectrometric (MS) detection can provide both qualitative and quantitative information at the same time on the assessment of undesirable substances in food and feed [20].

Mycotoxin toxicity must be evaluated to carry out an adequate risk assessment. In this field, some in silico approaches can provide precise information on the toxicokinetics and the toxicity of some less studied mycotoxins in both food and feed. Thus, in the present study, the oral toxicity and other toxicological endpoints of identified mycotoxins were predicted by using the in silico webserver ProTox-II [21]. Within this context, the aim of this study was to determine the mycotoxin occurrence in edible tissues of Atlantic salmon (*Salmo salar*) using a multianalyte method consisting in liquid chromatography coupled to hybrid quadrupole time-of-flight mass spectrometry (LC/Q-TOF MS) and also to predict the potential toxicity of the identified mycotoxins by in silico approaches.

## 2. Results and Discussion

### 2.1. Mycotoxin Identification by Non-Target Screening

In this study, LC/Q-TOF-MS was used for structural elucidation, identification, characterization and confirmation of the chemical formulas of mycotoxins due to its improved full-scan sensitivity, mass accuracy and resolving power compared to other equipment such as quadrupole mass spectrometers [22,23,24,25,26]. 

TOF analyzer allowed us to investigate the presence of 233 mycotoxins available in a wide list of validated compounds found in a homemade spectral library showing the presence of forty mycotoxins in analyzed salmon fillets (Table 1). To the best of our knowledge, this is the first study reporting the presence of these mycotoxins in fish from aquaculture farming directly purchased from supermarkets.

**Figure 1:**
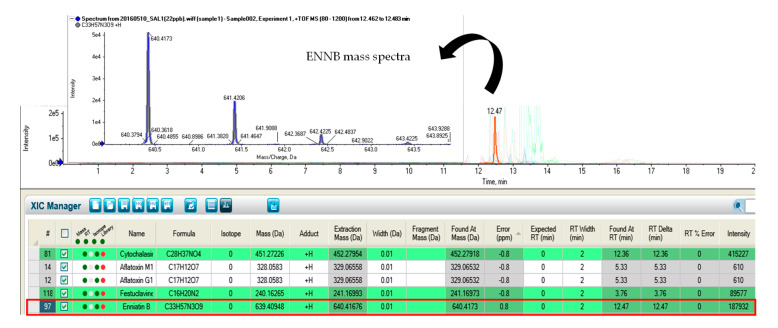
Chromatogram showing enniatin B (ENNB) identification by LC-Q-TOF-MS.

Although the presence of these fungal metabolites has been scarcely reported in feedstuffs and animal derived products, some of them are common contaminants of cereal-based foodstuffs from wheat and corn [26], such as enniatins (ENNA, ENNA1, ENNB, ENNB1 and ENNB2) (Figure 1) and fusaproliferin (FUS).

On the other hand, other less reported mycotoxins in feedstuffs were detected, mainly anisomycin, cytochalasin J (CJ), mycophenolic acid (MPA), ophiobolin A (OA) and B (OB), rugulosin and penicillic acid (PA), among others. 

Some of the mycotoxins identified in this study, namely chanoclavine, sulochrin, festuclavine, MPA, FB2 and ENNs, have been reported mainly in bread samples [27,28], while other mycotoxins have been also identified in feed and raw materials used in feed manufacture, such as MPA, cyclopiazonic acid, PA, radicicol, rugulosin and CJ, as evidenced by Streit et al. [7]. For instance, the method developed by Rundberget and Wilkins [29] allowed the simultaneous determination of MPA together with other less reported mycotoxins in both food and feed, while Sulyok et al. [30] were able to detect 15 mycotoxins in wheat and maize kernels similar to those found in this study. Moreover, Zhao et al. [17], reported that mycotoxin contamination in feed directly influences the presence of mycotoxins in animal derived products, as they can be retained in organs and edible tissues after metabolization and can be also excreted in some by-products. These results allow us to conclude that these mycotoxins could be present in edible tissues of animals who consume those contaminated feedstuffs, as observed in our study [25]. 

Recent surveys have revealed that diverse fish species in European aquaculture are commonly exposed to *Fusarium* mycotoxins in feed [8]. Emerging *Fusarium* mycotoxins were previously detected by our research team [9], and diverse studies have identified mainly AFB1 and/or its metabolites in different organs and tissues from exposed fish [10,31,32,33]. Nácher-Mestre et al. (2013) [25] applied a screening method to feed and fish fillets performed by UHPLC/Q-TOF-MS, confirming the presence of FB2 and ZEA in feed samples; however, no mycotoxin contamination was detected in fish fillets. 

**Figure 2:**
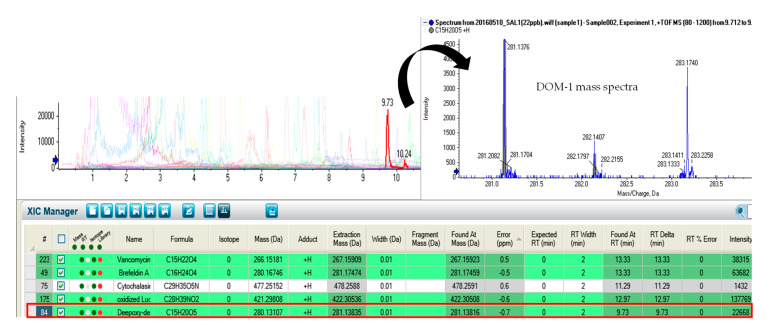
Chromatogram showing the identification of deepoxy-deoxynivalenol (DOM-1).

In a subsequent study, these authors evaluated the mycotoxin carry-over of aflatoxins (AFs), trichothecenes (TCs) and FBs, from feed to fish fillets in Atlantic salmon (*Salmo Salar*) and gilthead sea bream (*Sparus aurata*) [8], concluding that no mycotoxin carry-over was found in analyzed samples. Conversely, Guan et al. [32] evaluated DON occurrence and described the TC transformation by deacetylation and/or de-epoxidation reactions in different fish species. This fact is in accordance with our findings, where DON was not detected, but deepoxy-deoxynivalenol (DOM-1), obtained from DON de-epoxidation, was present in salmon fillets analyzed (Figure 2). This could be explained because DON is rapidly metabolized and its retention and accumulation in animal tissues is generally low [34]. These findings were also supported by Tola et al. [35], who described that DOM-1 was formed by DON de-epoxidation and deacetylation by microorganisms from the digestive tract in fish species. In addition, other assays have revealed that microbes in the digestive tract of brown bullhead (*Ameiurus nebulosus*), brown trout (*Salmo trutta*), pink salmon (*Oncorhynchus gorbuscha*) and other fish species were capable of transforming DON into DOM-1, while hepatic microsomes in the liver of common carp (*Cyprinus carpio*) were able to transform DON into deoxynivalenol 3 glucuronide (DON-3-glc). Moreover, according to the study reported by Bernhoft et al. (2017) [12], DON was metabolized in the liver of Atlantic salmon (*Salmo salar*) exposed to DON contaminated feed, resulting in the formation of DON-3glc. In their study, DON residues were detected in all tissues; however, when compared to terrestrial species, it can be observed that in Atlantic salmon the elimination of DON could be considerably slower.

Within the identified molecules, some of them corresponded to antibiotics, namely tetracyclines and β-lactams. The presence of these veterinary drug residues in edible tissues can be explained by their use in the treatment of food-producing animals. In animal production, when veterinary drugs are used, it is mandatory to respect a withdrawal period before the slaughter of animals intended for human consumption to avoid the presence of their residues in animal by-products, which can suppose a risk for consumers in terms of allergy and antibioresistance. In 2017, the World Health Organization (WHO) recommended reducing antibiotic use in animals used in the food industry, due to the increasing risk of antibiotic resistant bacteria, concluding that animals that require antibiotics should be treated with antibiotics that pose the smallest risk to human health. Some studies have established connections between antibiotic resistant infections and food-producing animals. Thus, it must be pointed out that antibiotic use in farm animals contributes to the overall problem of antibiotic resistance and thus poses an additional hazard of this animal by-products for consumers. 

Furthermore, some compounds from the Penicillin family have been identified. Allergic reactions to penicillins have been commonly reported even at therapeutic doses. This fact highlights the importance of avoiding the presence of these undesirable compounds in animal origin products which can produce serious allergic reactions to consumers. 

### 2.2. In Silico Toxicity Prediction 

Most of the identified mycotoxins in the present study have not been commonly reported in scientific literature. Thus, little information on their toxicity is available. For this reason, *in silico* prediction methods were used in this survey to predict the toxicity of detected and identified mycotoxins.

#### ProTox-II

The oral toxicity prediction data provided by ProTox-II are based in 2D similarity and the recognition of toxic fragments. Results are expressed as LD50 (mg/kg). In Table 2, the predicted LD50 and the corresponding toxicity class for each identified mycotoxin are shown. In material and methods section, the characteristics to classify the substances within different toxicity groups are described.

It should be highlighted that, according to the obtained predictions, ENNB and ENNB2 showed a predicted LD50 of 3 mg/kg, both with a 100% of average similarity and prediction accuracy. Thus, the assigned toxicity class was 1. Therefore, special attention should be paid to these mycotoxins due to their predicted toxicity, which is comparable to that of T-2 Toxin (Table 2), the latter being a toxic fungal metabolite with the lowest tolerable daily intake (TDI) within the *Fusarium* mycotoxins [36]. Regarding mycotoxins classified in category 2 (LD50 between 5 and 50 mg/kg), we found DOM-1, which showed a predicted LD50 of 34 mg/kg. In the case of oxidized luol, no prediction results could be obtained due to its chemical structure.

Using the ProTox-II web server, the organ toxicity (hepatotoxicity) can be also predicted, which was evaluated for different identified mycotoxins as the liver is the organ where mycotoxins are metabolized. In Table 3, the results obtained regarding the organ toxicity and the calculated prediction values for diverse toxicological endpoints using the ProTox-II web server are reported.

Regarding the organ toxicity, results obtained showed that cyclopenin, phomopsin A and tetracyclin were predicted as hepatotoxic. On the other hand, regarding the different toxicity endpoints evaluated, some mycotoxins were shown to be carcinogenic, immunotoxic, mutagenic and/or cytotoxic. Both fumigaclavine A and T-2 toxin were predicted as carcinogenic, immunotoxic and mutagenic substances, while curvularin, FB2, ophiobolin B, radicicol, rugulosin and vancomycin were predicted as carcinogenic and immunotoxic.

Within the toxicological endpoints, carcinogenicity and mutagenicity are relevant parameters to evaluate and to assess the toxic potential of different substances [36]. In this survey, Chanoclavine 56, cyclopenin, DOM-1, dihidrolysergol, festuclavine and methysergide were predicted as mutagenic compounds, while fumigaclavine A and T-2 toxin were predicted as both carcinogenic and mutagenic compounds (Table 3).

ENN B and ENNB2 were predicted as cytotoxic mycotoxins, a fact already reported in different studies performed by in vitro assays in different cell cultures [37,38]. The same occurs in the case of ophiobolin B (predicted as carcinogenic and immunotoxic), which has been described as toxic to animals in in vivo toxicity assays in mice [39].

In Table 4 and Table 5, the prediction results obtained for the toxicological pathways, nuclear receptor signaling pathways and stress response pathways are reported, respectively. According to the Tox21 Consortium, chemical compounds might have the potential to disrupt processes in the human body that may lead to negative health effects [21]. Regarding the nuclear receptor signaling pathway, seven different pathways were assessed. The computational estimations revealed that curvularin and sulochrin could interact with the estrogen receptor alpha (ER), FK 506 was active to interact with aromatase receptor and methysergide could interact with the aryl hydrocarbon receptor (AhR). Regarding the stress response pathways, five diverse assays were assessed by in silico approaches. Computational predictions indicated that special attention should be paid to curvularin, which showed to be active to interact with the nuclear factor (erythroid-derived 2-like 2/antioxidant responsive element (nrf2/ARE), heat shock response element (HSE), mitochondrial membrane potential (MMP) and phosphoprotein p53 (tumor supressor).

## 3. Materials and Methods 

### 3.1. Samples

Norwegian Atlantic salmon (*Salmo salar*) (10 samples) from aquaculture farming were acquired from different supermarkets located in the metropolitan area of Valencia (Spain) and analyzed for mycotoxin content determination. Samples were acquired in individual packages at different markets within one month in 2016, and they came from different producers and batches. All samples were stored in a dark and dry place at −20 °C until analysis. After their packages had been opened, they were analyzed within the same day. These samples were first analyzed by LC-MS/MS LIT, and results showing ENN contents were reported in a previous study [40]. The results showed some unidentified peaks; thus, those samples were analyzed by LC-Q-TOF-MS in order to identify those compounds by exact mass.

### 3.2. Mycotoxin Extraction and LC-Q-TOF-MS Analysis

The mycotoxin extraction used was carried out according to the method previously reported by Tolosa et al. [9]. For chromatographic separation, an Agilent 1290 HPLC system (Agilent, Santa Clara, CA, USA) with an Acquity UHPLC BEH C18 analytical column (50 × 2.1 mm and 1.7 μm particle size) (Waters) at a flow rate of 350 μL/min was employed. The column temperature was set to 60 °C, and the injected volume was 10 µL, while the mobile phase consisted in water (0.15 mM ammonium formate) and MeOH 0.1% formic acid. The percentage of organic modifier (B) was changed linearly as follows: 0 min, 5%; 2 min, 25%; 13 min, 100%; 15 min, 100%; 15.1 min, 5%; 25 min, 5%.

A hybrid quadrupole-orthogonal acceleration-TOF mass spectrometer (AB SCIEX TripleTOF™ 5600 LC/MS/MS System, Ontario, Canada), with an orthogonal Z-spray-ESI interface operating in positive ion mode, was used. The data acquisition was performed in positive mode, and mode mass spectra were acquired in a scan range from 100 to 1000 m/z, with a resolving power of 10,000 full width at half maximum (FWHM) mass resolution at *m*/*z* 556.2771. For automated accurate mass measurement, an external calibrant delivery system (CDS) which infuses calibration solution was used prior to sample injection. The MS was carried out using an IDA acquisition method with the survey scan type (TOF-MS) and the dependent scan type (product ion) using 50V of collision energy (CE). Data were qualitatively evaluated using the PeakViewTM software (AB Sciex, Ontario, Canada).

Ion source parameters were as follows: cone voltage 25 V, capillary voltage 3.5 kV, desolvation temperature 500 °C, interface temperature 450 °C and source temperature 120 °C. Ion Spray Voltage (ISVF) was 5500 and declustering potential, 120 V. The Ion source gas 1 (GC1) and 2 (GC2) were 40 psi.

To promote ion-source fragmentation in MS^2^ experiments, an acquisition function with medium CE of 50 V was applied using argon as the collision gas (99.995%; Praxair, Madrid, Spain). 

### 3.3. Non-Targeted Suspect Screening (TOF)

Mass spectrometry (MS) is among the most employed methods for structure elucidation, and high resolution MS is the method of choice for the identification of unknown masked mycotoxins in processed or unprocessed food [24]. In the non-target screening carried out in the present study, the compounds were identified by the exact *m*/*z* ion in chromatograms by searching in a database containing the empirical formula, the RT, isotopic abundance, number of double bonds and product ion mass spectra.

### 3.4. In Silico Prediction Methods

To carry out the prediction by in silico methods, the ProTox-II platform was used [21,41]. The only essential information to carry out the prediction is the chemical structure or the Pubchem-name of the molecule. The ProTox-II platform is divided into a five different classification steps: (1) acute toxicity (oral toxicity model with six different toxicity classes); (2) organ toxicity (1 model); (3) toxicological endpoints (4 models); (4) toxicological pathways (12 models) and (5) toxicity targets (15 models). 

ProTox-II incorporates molecular similarity, fragment propensities, most frequent features (fragment similarity-based CLUSTER cross-validation) and machine-learning, based a total of 33 models for the prediction of various toxicity endpoints such as acute toxicity, hepatotoxicity, cytotoxicity, carcinogenicity, mutagenicity, immunotoxicity, adverse outcomes pathways (Tox21) and toxicity targets.

#### 3.4.1. Acute Oral Toxicity Prediction

Substances are classified into different toxicity classes, depending on the LD50 (mg/kg body weight), which are defined according to the globally harmonized system of classification in labelling of chemicals (GHS):

- Class I: fatal if swallowed (LD50 ≤ 5 mg/kg);
- Class II: fatal if swallowed (5 mg/kg < LD50 ≤ 50 mg/kg);
- Class III: toxic if swallowed (50 mg/kg < LD50 ≤ 300 mg/kg);
- Class IV: harmful if swallowed (300 mg/kg < LD50 ≤ 2000 mg/kg);
- Class V: may be harmful if swallowed (2000 mg/kg < LD50 ≤ 5000 mg/kg).

#### 3.4.2. Toxicity Endpoint and Organ Toxicity Prediction

The same in silico prediction tool (ProTox-II) was employed for the prediction of various toxicity endpoints; namely hepatotoxicity, cytotoxicity, carcinogenicity, mutagenicity and immunotoxicity. The predictive models are based on data from both in vitro (e.g., Tox21 assays, Ames bacterial mutation assays, hepG2 cytotoxicity assays and immunotoxicity assays) and in vivo assays (e.g., carcinogenicity, hepatotoxicity).

#### 3.4.3. Toxicological Pathways

Two types of target-pathway-based models are implemented In ProTox-II: (i) Nuclear Receptor Signaling Pathways (7 pathway assays shown in Table 4) and (ii) Stress Response Pathways (5 pathway assays shown in Table 5) [41].

This approach is based in the fact that a chemical compound can activate or inhibit a receptor or an enzyme when it interacts with them, resulting in a perturbation in diverse biological pathways, thereby disrupting the cellular process and causing cell death. The main purpose of the initiative is to prioritize substances for further in-depth toxicological evaluation as well as to identify some mechanisms for further investigation such as disease-associated pathways. Thus, by applying this computational prediction tool, it is possible to test quickly and efficiently whether certain chemical compounds have the potential to disrupt processes in the human body that may lead to adverse health effects.

## 4. Conclusions

From the results obtained, it is possible to conclude that the use of a multiclass screening methodology was shown to be effective for the identification of 40 mycotoxins in edible salmon tissues from aquaculture using a homemade database with 233 compounds. Screening selectivity was supported by accurate mass measurements provided by the Q-TOF-MS technique. It is the first time that these 40 mycotoxins have been identified and documented in farmed fish, as they had previously only been found in different cereal samples. The explanation for the appearance of these mycotoxins in farmed fish is the inclusion of cereals with mycotoxins as raw material in the feed during the processing and manufacturing processes, which results in the carryover of the feed to the edible parts of the fish. Furthermore, a metabolite formed through de-epoxidation of DON (DOM-1) was detected in salmon tissues. Therefore, it is necessary to ensure that farmed fish for human consumption is free of contaminants or contains concentrations below the maximum limits established for legislated mycotoxins. In light of these findings, the potential health risk associated with eating mycotoxin-contaminated fish should attract the public’s attention, as these products are an important part of the daily diet in combination with other foods. These results are supported by the fact that some of the detected mycotoxins showed a low LD50 using in silico approaches. However, the next purpose is to confirm these findings achieved through in silico predictions with in vitro techniques to corroborate the results obtained.

## Figures and Tables

**Table 1:** Identified mycotoxins in salmon samples.

Mycotoxins and Other Fungal Metabolites	Elemental Composition	Exact Mass (*m*/*z*) **	RT * (min)
2-amino-14,16-dimethyloctadecan-3-ol	C20H43NO	314.3417	13.42
Anisomycin	C14H19NO4	266.1387	1.42
Chanoclavine	C16H20N20	257.1648	2.66
Curvularin	C16H20O5	292.1310	8.29
Cyclopenin	C17H14N2O3	295.1077	11.53
Cyclopiazonic acid	C20H20N2O3	337.1547	6.19
Cytochalasin J	C28H37NO4	452.2795	12.36
Deoxybrevianamide E	C21H25N3O2	352.2020	4.86
Deepoxy-deoxynivalenol	C15H20O5	281.1376	9.73
Dihydrolysergol	C16H20N2O	257.1648	2.66
Enniatin A	C36H63N3O9	682.4637	13.28
Enniatin A1	C35H61N3O9	668.4481	13.04
Enniatin B	C33H57N3O9	640.4168	12.47
Enniatin B1	C34H59N3O9	654.4324	12.78
Enniatin B2	C32H55N3O9	626.4011	12.50
Festuclavine	C16H20N2	241.1699	3.80
FK 506	C44H69NO12	804.4893	6.33
Fumigaclavine A	C18H22N2O2	299.1754	11.49
Fumitremorgin C	C22H25N3O3	380.1969	4.33
Fumonisin B2	C34H59NO14	706.4008	13.20
Fusaproliferin	C27H40O5	445.2949	13.19
Fusidic acid	C31H48O6	517.3524	14.40
Methysergide	C21H27N3O2	354.2176	12.20
Mycophenolic acid	C17H20O6	321.1333	2.97
Myriocin	C21H39NO6	402.2850	7.68
Ophiobolin A	C25H36O4	401.2686	13.28
Ophiobolin B	C25H38O4	403.2843	13.33
Oxidized luol	C28H39NO2	422.3054	12.97
Paspaline	C32H39NO4	502.2952	12.57
Penicillic acid	C16H18N2O5S	351.1009	11.26
Penicillin G	C27H33NO6	468.2381	11.06
Penicillin V	C37H44O6NCl	634.2930	9.79
Phomopsin A	C22H25NO8	432.1653	10.55
Pseurotin A	C16H24O6	313.1646	8.54
Radicicol	C22H23N5O2	390.1925	8.59
Rugulosin	C29H38O8	515.2639	13.66
Sulochrin	C24H34O9	467.2276	4.35
T-2 Toxin	C22H30N4O4	415.2340	7.74
Tetracycline	C17H24O4	293.1747	13.35
Vancomycin	C15H22O4	267.1591	12.92

* RT retention time. ** Mycotoxins were detected as protonated ions [M + H]^+^.

**Table 2:** Acute Oral Toxicity prediction obtained by using ProTox-II web server.

Mycotoxin	Oral Toxicity Prediction Results			
	Predicted LD50 (mg/kg)	Predicted Toxicity Class	Average Similarity (%)	Prediction Accuracy (%)
2-amino-14,16-dimethyloctadecan-3-ol	3500	5	100	100
Alamethicin F30	80	3	100	100
Anisomycin	72	3	100	100
Chanoclavine	110	3	66	68
Curvularin	450	4	62	68
Cyclopenin	2200	5	58	67
Cyclopiazonic acid	93	3	67	68
Cytochalasin J	400	4	66	68
Deepoxy-deoxynivalenol	**34**	**2**	**89**	**71**
Deoxybrevianamide E	1000	4	67	68
Dihydrolysergol	110	3	90	73
Enniatin A	1600	4	76	69
Enniatin A1	1600	4	76	69
Enniatin B	**3**	**1**	**100**	**100**
Enniatin B1	1600	4	76	69
Enniatin B2	**3**	**1**	**100**	**100**
Festuclavine	110	3	95	73
FK 506	134	3	100	100
Fumigaclavine A	800	4	82	71
Fumitremorgin C	72	3	100	100
Fumonisin B2	4280	5	68	68
Fusaproliferin	5000	5	71	69
Fusidic acid	841	4	100	100
Methysergide	200	3	100	100
Mycophenolic acid	352	4	100	100
Myriocin	300	3	100	100
Ophiobolin A	238	3	100	100
Ophiobolin B	238	**3**	72	69
Oxidized luol	-	-	-	-
Paspaline	374	4	73	69
Penicillic acid	600	4	100	100
Penicillin G	1000	4	100	100
Penicillin V	1040	4	100	100
Phomopsin A	400	4	56	67
Pseurotin A	134	3	49	54
Radicicol	300	3	100	100
Rugulosin	220	3	53	67
Sulochrin	690	4	61	68
T-2 Toxin	**3**	**1**	**100**	**100**
Tetracycline	678	4	100	100
Vancomycin	300	3	100	100

**Table 3:** Organ toxicity and toxicological endpoints predicted activity calculated using the ProTox-II web server.

Mycotoxin	Classification				
	Organ Toxicity (% Probability)	Toxicity Endpoint (% Probability)			
	Hepatotoxicity	Carcinogenicity	Immunotoxicity	Mutagenicity	Cytotoxicity
2-amino-14,16-dimethyloctadecan-3-ol	Inactive (74)	Inactive (50)	Inactive (97)	Inactive (94)	Inactive (71)
Alamethicin F30	Inactive (97)	Inactive (57)	Inactive (99)	Inactive (85)	Inactive (78)
Anisomycin	Inactive (89)	Inactive (73)	Inactive (66)	Inactive (78)	Inactive (63)
Chanoclavine	Inactive (67)	Inactive (74)	Inactive (99)	**Active (56)**	Inactive (59)
Curvularin	Inactive (82)	**Active (65)**	**Active (57)**	Inactive (97)	Inactive (75)
Cyclopenin	**Active (56)**	Inactive (58)	Inactive (96)	**Active (52)**	Inactive (53)
Cyclopiazonic acid	Inactive (63)	Inactive (62)	Inactive (73)	Inactive (52)	Inactive (58)
Cytochalasin J	Inactive (68)	Inactive (54)	**Active (98)**	Inactive (72)	Inactive (75)
Deepoxy-deoxynivalenol	Inactive (80)	Inactive (77)	Inactive (64)	**Active (50)**	Inactive (70)
Deoxybrevianamide E	Inactive (81)	Inactive (61)	**Active (82)**	Inactive (63)	Inactive (70)
Dihydrolysergol	Inactive (92)	Inactive (68)	Inactive (95)	**Active (74)**	Inactive (69)
Enniatin A	Inactive (70)	Inactive (63)	Inactive (88)	Inactive (67)	Inactive (51)
Enniatin A1	Inactive (70)	Inactive (63)	Inactive (88)	Inactive (67)	Inactive (51)
Enniatin B	Inactive (73)	Inactive (66)	Inactive (97)	Inactive (64)	**Active (56)**
Enniatin B1	Inactive (70)	Inactive (63)	Inactive (88)	Inactive (67)	Inactive (51)
Enniatin B2	Inactive (72)	Inactive (66)	Inactive (81)	Inactive (65)	**Active (59)**
Festuclavine	Inactive (89)	Inactive (74)	Inactive (92)	**Active (93)**	Inactive (64)
FK 506	Inactive (87)	Inactive (50)	**Active (99)**	Inactive (70)	Inactive (64)
Fumigaclavine A	Inactive (83)	**Active (51)**	**Active (63)**	**Active (50)**	Inactive (65)
Fumitremorgin C	Inactive (89)	Inactive (73)	Inactive (66)	Inactive (78)	Inactive (63)
Fumonisin B2	Inactive (78)	**Active (74)**	**Active (52)**	Inactive (100)	Inactive (71)
Fusaproliferin	Inactive (90)	Inactive (62)	Inactive (95)	Inactive (87)	Inactive (71)
Fusidic acid	Inactive (73)	Inactive (52)	**Active (99)**	Inactive (87)	Inactive (63)
Methysergide	Inactive (96)	Inactive (61)	Inactive (73)	**Active (52)**	Inactive (89)
Mycophenolic acid	Inactive (85)	Inactive (58)	**Active (80)**	Inactive (93)	Inactive (88)
Myriocin	Inactive (85)	Inactive (59)	Inactive (99)	Inactive (90)	Inactive (71)
Ophiobolin A	Inactive (76)	Inactive (52)	**Active (98)**	Inactive (75)	Inactive (73)
Ophiobolin B	Inactive (80)	**Active (53)**	**Active (92)**	Inactive (72)	Inactive (72)
Oxidizedluol	-	-	-	-	-
Paspaline	Inactive (68)	Inactive (73)	**Active (95)**	Inactive (73)	Inactive (78)
Penicillic acid	Inactive (69)	Inactive (75)	Inactive (99)	Inactive (52)	Inactive (67)
Penicillin G	Inactive (87)	Inactive (83)	Inactive (99)	Inactive (97)	Inactive (60)
Penicillin V	Inactive (91)	Inactive (81)	Inactive (99)	Inactive (95)	Inactive (55)
Phomopsin A	**Active (55)**	**Active (53)**	Inactive (96)	Inactive (53)	Inactive (82)
Pseurotin A	Inactive (65)	Inactive (63)	**Active (62)**	Inactive (63)	Inactive (57)
Radicicol	Inactive (58)	**Active (53)**	**Active (96)**	Inactive (52)	Inactive (57)
Rugulosin	Inactive (64)	**Active (70)**	**Active (73)**	Inactive (94)	Inactive (69)
Sulochrin	Inactive (59)	Inactive (76)	Inactive (57)	Inactive (71)	Inactive (82)
T-2 Toxin	Inactive (85)	**Active (77)**	**Active (99)**	**Active (71)**	Inactive (64)
Tetracycline	**Active (58)**	Inactive (75)	**Active (99)**	Inactive (95)	Inactive (91)
Vancomycin	Inactive (58)	**Active (53)**	**Active (96)**	Inactive (52)	Inactive (57)

**Table 4:** Toxicological pathways: nuclear receptor signaling pathways predicted for detected mycotoxins.

Mycotoxin	Tox21 Nuclear Receptor Signaling Pathways (% Probability)						
	Aryl Hydrocarbon Receptor (AhR)	Androgen Receptor (AR)	Androgen Receptor Ligand Binding Domain (AR-LBD)	Aromatase	Estrogen Receptor Alpha (ER)	Estrogen Receptor Ligand Binding Domain (ER-LBD)	Peroxisome Proliferator Activated Receptor Gamma (PPAR-Gamma)
2-amino-14,16-dimethyloctadecan-3-ol	Inactive (98)	Inactive (99)	Inactive (99)	Inactive (99)	Inactive (88)	Inactive (99)	Inactive (98)
Alamethicin F30	Inactive (96)	Inactive (97)	Inactive (99)	Inactive (97)	Inactive (93)	Inactive (98)	Inactive (95)
Anisomycin	Inactive (93)	Inactive (95)	Inactive (99)	Inactive (94)	Inactive (90)	Inactive (94)	Inactive (99)
Chanoclavine	**Active (60)**	Inactive (90)	Inactive (88)	Inactive (79)	Inactive (78)	Inactive (96)	Inactive (92)
Curvularin	Inactive (85)	Inactive (97)	Inactive (98)	Inactive (82)	**Active (95)**	**Active (94)**	Inactive (94)
Cyclopenin	Inactive (79)	Inactive (91)	Inactive (99)	Inactive (80)	Inactive (90)	Inactive (97)	Inactive (94)
Cyclopiazonic acid	Inactive (80)	Inactive (92)	Inactive (97)	Inactive (79)	Inactive (86)	Inactive (94)	Inactive (94)
Cytochalasin J	Inactive (88)	Inactive (91)	Inactive (95)	Inactive (81)	Inactive (78)	Inactive (91)	Inactive (94)
Deepoxy-deoxynivalenol	Inactive (94)	Inactive (92)	Inactive (85)	Inactive (81)	Inactive (83)	Inactive (97)	Inactive (95)
Deoxybrevianamide E	Inactive (82)	Inactive (94)	Inactive (97)	Inactive (82)	Inactive (89)	Inactive (98)	Inactive (84)
Dihydrolysergol	Inactive (50)	Inactive (93)	Inactive (95)	Inactive (87)	Inactive (88)	Inactive (98)	Inactive (99)
Enniatin A	Inactive (96)	Inactive (94)	Inactive (97)	Inactive (97)	Inactive (90)	Inactive (97)	Inactive (97)
Enniatin A1	Inactive (96)	Inactive (94)	Inactive (97)	Inactive (97)	Inactive (90)	Inactive (97)	Inactive (97)
Enniatin B	Inactive (97)	Inactive (94)	Inactive (96)	Inactive (97)	Inactive (88)	Inactive (96)	Inactive (98)
Enniatin B1	Inactive (96)	Inactive (94)	Inactive (97)	Inactive (97)	Inactive (90)	Inactive (97)	Inactive (97)
Enniatin B2	Inactive (97)	Inactive (95)	Inactive (97)	Inactive (97)	Inactive (89)	Inactive (96)	Inactive (98)
Festuclavine	Inactive (52)	Inactive (97)	Inactive (96)	Inactive (91)	Inactive (88)	Inactive (98)	Inactive (96)
FK 506	Inactive (99)	Inactive (99)	Inactive (99)	**Active (79)**	Inactive (82)	Inactive (91)	Inactive (95)
Fumigaclavine A	Inactive (50)	Inactive (94)	Inactive (96)	Inactive (90)	Inactive (91)	Inactive (97)	Inactive (97)
Fumitremorgin C	Inactive (93)	Inactive (95)	Inactive (99)	Inactive (94)	Inactive (90)	Inactive (94)	Inactive (99)
Fumonisin B2	Inactive (98)	Inactive (96)	Inactive (99)	Inactive (99)	Inactive (85)	Inactive (96)	Inactive (97)
Fusaproliferin	Inactive (97)	Inactive (89)	Inactive (81)	Inactive (95)	Inactive (77)	Inactive (98)	Inactive (97)
Fusidic acid	Inactive (99)	Inactive (65)	Inactive (63)	Inactive (96)	Inactive (71)	Inactive (81)	Inactive (99)
Methysergide	**Active (100)**	Inactive (91)	Inactive (90)	Inactive (98)	Inactive (98)	Inactive (99)	Inactive (99)
Mycophenolic acid	Inactive (87)	Inactive (96)	Inactive (90)	Inactive (71)	Inactive (64)	Inactive (89)	Inactive (91)
Myriocin	Inactive (99)	Inactive (98)	Inactive (97)	Inactive (99)	Inactive (93)	Inactive (97)	Inactive (99)
Ophiobolin A	Inactive (97)	Inactive (83)	Inactive (75)	Inactive (63)	Inactive (85)	Inactive (94)	Inactive (95)
Ophiobolin B	Inactive (97)	Inactive (85)	Inactive (82)	Inactive (87)	Inactive (81)	Inactive (92)	Inactive (98)
Oxidizedluol	-	-	-	-	-	-	-
Paspaline	Inactive (80)	Inactive (89)	Inactive (85)	Inactive (78)	Inactive (81)	Inactive (89)	Inactive (88)
Penicillic acid	Inactive (99)	Inactive (99)	Inactive (98)	Inactive (96)	Inactive (95)	Inactive (97)	Inactive (93)
Penicillin G	Inactive (96)	Inactive (97)	Inactive (99)	Inactive (99)	Inactive (97)	Inactive (99)	Inactive (98)
Penicillin V	Inactive (97)	Inactive (96)	Inactive (99)	Inactive (98)	Inactive (96)	Inactive (98)	Inactive (97)
Phomopsin A	Inactive (53)	Inactive (95)	Inactive (96)	Inactive (85)	Inactive (80)	Inactive (89)	Inactive (92)
Pseurotin A	Inactive (88)	Inactive (97)	Inactive (99)	Inactive (88)	Inactive (88)	Inactive (95)	Inactive (94)
Radicicol	Inactive (75)	Inactive (94)	Inactive (94)	Inactive (84)	Inactive (81)	Inactive (85)	Inactive (85)
Rugulosin	Inactive (74)	Inactive (92)	Inactive (91)	Inactive (79)	Inactive (54)	Inactive (62)	Inactive (85)
Sulochrin	Inactive (60)	Inactive (93)	Inactive (100)	Inactive (92)	**Active (74)**	Inactive (68)	Inactive (95)
T-2 Toxin	Inactive (96)	Inactive (87)	Inactive (86)	Inactive (85)	Inactive (74)	Inactive (97)	Inactive (91)
Tetracycline	Inactive (87)	Inactive (99)	Inactive (98)	Inactive (98)	Inactive (98)	Inactive (99)	Inactive (99)
Vancomycin	Inactive (75)	Inactive (94)	Inactive (94)	Inactive (84)	Inactive (81)	Inactive (85)	Inactive (85)

**Table 5:** Toxicological pathways: stress response pathways predicted for detected mycotoxins.

Mycotoxin	Nuclear Factor (Erythroid-Derived 2-Like 2/Antioxidant Responsive Element) (nrf2/ARE)	Heat Shock Factor Response Element (HSE)	Mitochondrial Membrane Potential (MMP)	Phosphoprotein (Tumor Supressor) p53	ATPase Family AAA Domain Containing Protein 5 (ATAD5)
2-amino-14,16-dimethyloctadecan-3-ol	Inactive (96)	Inactive (96)	Inactive (95)	Inactive (99)	Inactive (99)
Alamethicin F30	Inactive (98)	Inactive (98)	Inactive (94)	Inactive (94)	Inactive (97)
Anisomycin	Inactive (97)	Inactive (97)	Inactive (93)	Inactive (96)	Inactive (98)
Chanoclavine	Inactive (92)	Inactive (92)	Inactive (64)	Inactive (85)	Inactive (88)
Curvularin	**Active (79)**	**Active (79)**	**Active (94)**	**Active (64)**	Inactive (97)
Cyclopenin	Inactive (91)	Inactive (91)	Inactive (66)	Inactive (73)	Inactive (85)
Cyclopiazonic acid	Inactive (85)	Inactive (85)	Inactive (55)	Inactive (74)	Inactive (96)
Cytochalasin J	Inactive (86)	Inactive (86)	Inactive (69)	Inactive (74)	Inactive (90)
Deepoxy-deoxynivalenol	Inactive (90)	Inactive (90)	Inactive (78)	Inactive (87)	Inactive (90)
Deoxybrevianamide E	Inactive (93)	Inactive (93)	Inactive (77)	Inactive (77)	Inactive (96)
Dihydrolysergol	Inactive (95)	Inactive (95)	Inactive (87)	Inactive (96)	Inactive (98)
Enniatin A	Inactive (96)	Inactive (94)	Inactive (97)	Inactive (97)	Inactive (90)
Enniatin A1	Inactive (96)	Inactive (94)	Inactive (97)	Inactive (97)	Inactive (90)
Enniatin B	Inactive (97)	Inactive (94)	Inactive (96)	Inactive (97)	Inactive (88)
Enniatin B1	Inactive (96)	Inactive (94)	Inactive (97)	Inactive (97)	Inactive (90)
Enniatin B2	Inactive (97)	Inactive (95)	Inactive (97)	Inactive (97)	Inactive (89)
Festuclavine	Inactive (52)	Inactive (97)	Inactive (96)	Inactive (91)	Inactive (88)
FK 506	Inactive (99)	Inactive (99)	Inactive (99)	Active (79)	Inactive (82)
Fumigaclavine A	Inactive (50)	Inactive (94)	Inactive (96)	Inactive (90)	Inactive (91)
Fumitremorgin C	Inactive (93)	Inactive (95)	Inactive (99)	Inactive (94)	Inactive (90)
Fumonisin B2	Inactive (98)	Inactive (96)	Inactive (99)	Inactive (99)	Inactive (85)
Fusaproliferin	Inactive (97)	Inactive (89)	Inactive (81)	Inactive (95)	Inactive (77)
Fusidic acid	Inactive (99)	Inactive (65)	Inactive (63)	Inactive (96)	Inactive (71)
Methysergide	**Active (100)**	Inactive (91)	Inactive (90)	Inactive (98)	Inactive (98)
Mycophenolic acid	Inactive (87)	Inactive (96)	Inactive (90)	Inactive (71)	Inactive (64)
Myriocin	Inactive (99)	Inactive (98)	Inactive (97)	Inactive (99)	Inactive (93)
Ophiobolin A	Inactive (97)	Inactive (83)	Inactive (75)	Inactive (63)	Inactive (85)
Ophiobolin B	Inactive (97)	Inactive (85)	Inactive (82)	Inactive (87)	Inactive (81)
Oxidizedluol	-	-	-	-	-
Paspaline	Inactive (80)	Inactive (89)	Inactive (85)	Inactive (78)	Inactive (81)
Penicillic acid	Inactive (99)	Inactive (99)	Inactive (98)	Inactive (96)	Inactive (95)
Penicillin G	Inactive (96)	Inactive (97)	Inactive (99)	Inactive (99)	Inactive (97)
Penicillin V	Inactive (97)	Inactive (96)	Inactive (99)	Inactive (98)	Inactive (96)
Phomopsin A	Inactive (53)	Inactive (95)	Inactive (96)	Inactive (85)	Inactive (80)
Pseurotin A	Inactive (88)	Inactive (97)	Inactive (99)	Inactive (88)	Inactive (88)
Radicicol	Inactive (75)	Inactive (94)	Inactive (94)	Inactive (84)	Inactive (81)
Rugulosin	Inactive (74)	Inactive (92)	Inactive (91)	Inactive (79)	Inactive (54)
Sulochrin	Inactive (60)	Inactive (93)	Inactive (100)	Inactive (92)	**Active (74)**
T-2 Toxin	Inactive (96)	Inactive (87)	Inactive (86)	Inactive (85)	Inactive (74)
Tetracycline	Inactive (87)	Inactive (99)	Inactive (98)	Inactive (98)	Inactive (98)
Vancomycin	Inactive (75)	Inactive (94)	Inactive (94)	Inactive (84)	Inactive (81)
